# Nontranslocation follicular lymphoma mimicking infectious mononucleosis: a diagnostic challenge in a 39-year-old male

**DOI:** 10.1093/omcr/omag048

**Published:** 2026-04-28

**Authors:** Elinor Barsh, Jacob Keeling, Changlee S Pang, Ross Sattler, Kavanya Feustel, Alexander Enurah

**Affiliations:** Sky Ridge Medical Center Department of Internal Medicine, 10101 RidgeGate Pkwy, Lone Tree, CO 80124, United States; Sky Ridge Medical Center Department of Internal Medicine, 10101 RidgeGate Pkwy, Lone Tree, CO 80124, United States; CU Anschutz Department of Hematopathology, 12631 East 17th Avenue, Aurora, CO 80045, United States; Sky Ridge Medical Center Department of Internal Medicine, 10101 RidgeGate Pkwy, Lone Tree, CO 80124, United States; Sky Ridge Medical Center Department of Internal Medicine, 10101 RidgeGate Pkwy, Lone Tree, CO 80124, United States; Sky Ridge Medical Center Department of Internal Medicine, 10101 RidgeGate Pkwy, Lone Tree, CO 80124, United States

**Keywords:** B-cell dysfunction, malignancy, mononucleosis, Heterophile antibodies, false positives, delayed diagnosis, Hematology, lymphoma

## Abstract

Follicular lymphoma is an indolent B-cell non-Hodgkin lymphoma typically characterized by the t(14;18)(q32;q21) translocation resulting in B-cell lymphoma 2 (BCL2)/immunoglobulin heavy chain (IGH) fusion. Nontranslocation variants are rare and may present with atypical clinical features that complicate diagnosis. The patient’s presentation initially resembled infectious mononucleosis due to a false-positive monospot test; however, subsequent workup revealed follicular lymphoma lacking the canonical BCL2/IGH translocation. Extensive infectious, autoimmune, and nutritional evaluation was unrevealing. Cervical lymph node biopsy demonstrated follicular lymphoma with uniform BCL2 expression but no detectable BCL2/IGH fusion by Fluorescence in Situ Hybridization (FISH), confirming nontranslocation follicular lymphoma. The patient achieved complete remission after six cycles of obinutuzumab, cyclophosphamide, vincristine, and prednisone. This case illustrates a rare presentation of nontranslocation follicular lymphoma mimicking infectious mononucleosis and emphasizes the importance of reconsidering hematologic malignancy in patients with persistent mucocutaneous inflammation and incongruent laboratory findings.

## Introduction

Follicular lymphoma is the most common indolent non-Hodgkin lymphoma, accounting for approximately 20%–30% of adult lymphomas [1]. It is classically defined by the translocation t(14;18), juxtaposing the IGH locus with BCL2 gene, resulting in overexpression of the anti-apoptotic BCL2 protein and prolonged B-cell survival [1].

While most patients present with asymptomatic lymphadenopathy and follow an indolent course, variants lacking the BCL2/IGH fusion, known as nontranslocation follicular lymphoma, pose diagnostic challenges [2]. These retain BCL2 expression through alternative mechanisms and may mimic infectious or inflammatory conditions.

We describe a rare case of nontranslocation follicular lymphoma presenting with severe purulent stomatitis. This case highlights the diagnostic complexity of follicular lymphoma without the canonical translocation and the need to consider lymphoid malignancy in patients with persistent mucocutaneous inflammation and incongruent laboratory findings.

## Case report

A 39-year-old man with a mechanical mitral valve on warfarin presented after three months of purulent stomatitis, poor oral intake, and 35 kilograms weight loss. One month earlier, at an outside hospital, he had neutrophilic leukocytosis, cervical lymphadenopathy, and a positive monospot latex agglutination test. Computed tomography (CT) imaging ruled out abscess, and symptoms briefly resolved after a short steroid course.

**Figure 1:**
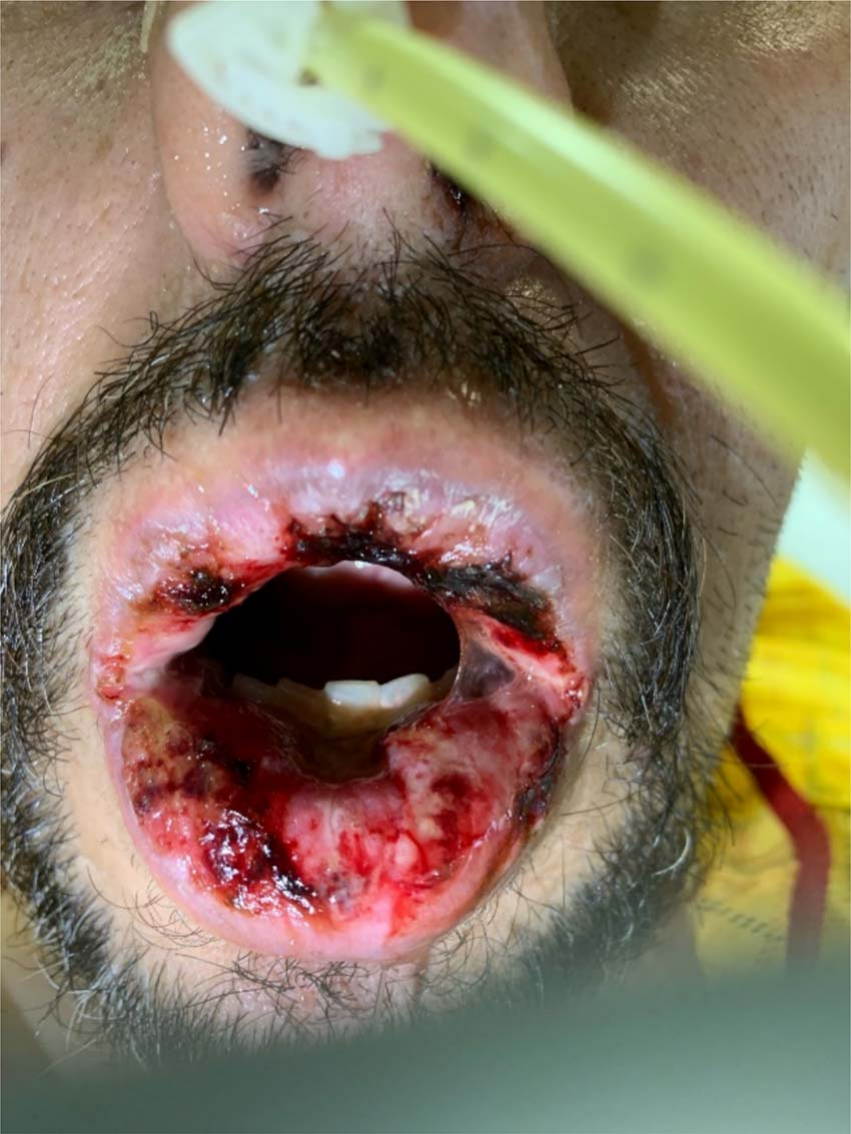
Patient’s purulent stomatitis was the main cause of weight loss and discomfort. These lesions produced upwards of 500ccs of sterile neutrophilic exudate daily that severely limited oral intake.

On admission three months later, he had painful stomatitis involving the gingiva overlying the mandibular alveolar processes, producing about 500 milliliters of purulent sputum daily (Fig. 1), and a 1.5-centimeter lymph node at level III of the left anterior cervical chain. Basic labs and head/neck CT were unremarkable. Nasogastric enteral nutrition and comprehensive diagnostic workup were initiated.

Differential diagnoses included infectious, autoimmune, and nutritional causes. A viral polymerase chain reaction and immunoglobulin panel that included herpes simplex virus 1 and 2, human immunodeficiency virus, cytomegalovirus, parvovirus, Epstein–Barr virus (EBV), and hepatitis A, B, and C were ordered on admission. Only EBV immunoglobulin G was positive; immunoglobulin M was negative. Penile lesions cultured confirmed *Candida albicans* balanitis and resolved with topical fluconazole. Gram stains and a potassium hydroxide preparation of oral drainage were repeatedly negative. Later, spontaneous purulent testicular lesions grew *Staphylococcus aureus*, treated with vancomycin then topical mupirocin.

Workup for vasculitis showed no anti-neutrophil antibodies or rheumatoid factor. Vitamin C deficiency was considered but serum levels were normal, and exam showed no perifollicular petechiae or hair abnormalities. Other micronutrient deficiencies were not evident. Given concurrent oral and genital lesions, Sweet syndrome and Behçet’s disease were briefly considered but deemed unlikely due to the purulent character of lesions, absence of pathergy, and prior culture-proven infections.

**Figure 2:**
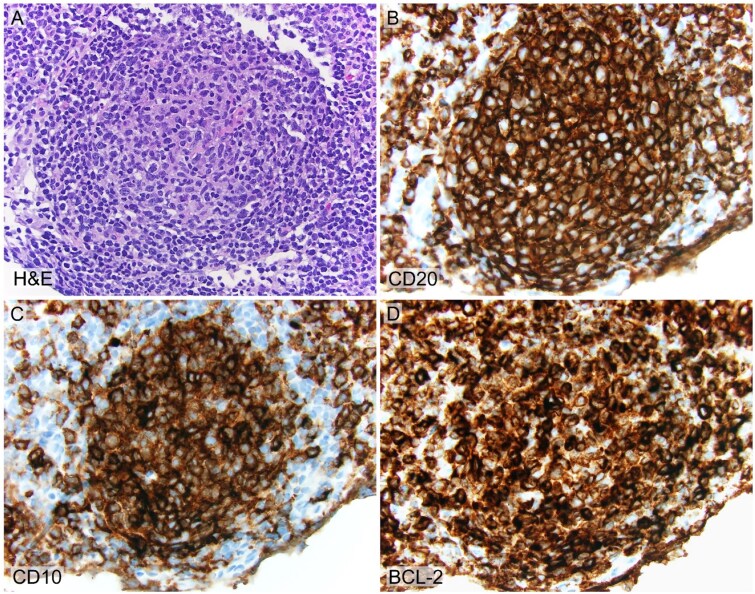
High-power view of nonpolarized neoplastic follicles composed of multiple centrocytes and fewer centroblasts with H&E (A) and immunohistochemistry for CD20 (B), CD10 (C) and BCL2 (C). Microscopic description of Fig. 2: The needle core biopsy showed multiple nonpolarized neoplastic follicles with attenuated mantle zones. The neoplastic follicles are composed of numerous centrocytes and fewer centroblasts with histologic grade 2 of 3. The neoplastic centrocytes and centroblasts are positive for CD20 (B-cell marker) and CD10 (germinal center marker) with aberrant coexpression of BCL2 (anti-apoptotic protein), consistent with follicular lymphoma.

**Figure 3:**
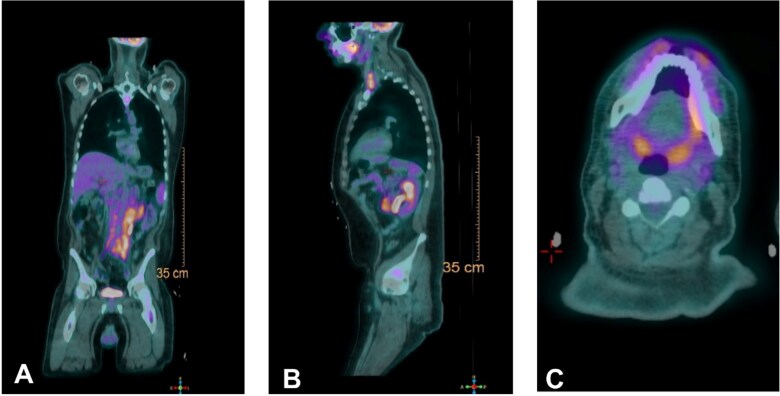
PET scan of patient with AP (A) lateral (B) and transverse plane of mandible (C) demonstrating widespread hypermetabolic lymphadenopathy noted in the neck, jaw, and retroperitoneum consistent with biopsy and patient presentation.

Needle core biopsy of the cervical lymph node revealed uniform B-cell populations organized in an architecture reminiscent of nodal follicles. BCL2 was uniformly overexpressed, consistent with follicular lymphoma. However, FISH analysis was negative for the BCL2/IGH fusion, confirming nontranslocation follicular lymphoma (Fig. 2). Positron emission tomography (PET) imaging revealed increased uptake in the biopsied node (standardized uptake value, SUV, max: 6.6) and retroperitoneal nodes (SUV max: 8.1) (Fig. 3).

The patient completed six cycles of obinutuzumab, cyclophosphamide, vincristine, and prednisone (doxorubicin omitted due to cardiac history). Follow-up PET/CT demonstrated Deauville 3 complete remission. He remains on maintenance obinutuzumab (12 cycles over 2 years) and is under rheumatologic evaluation for persistent stomatitis.

## Discussion

Follicular lymphoma is the most common indolent non-Hodgkin’s lymphoma in the United States, with 15 000–20 000 new cases annually [3]. The pathognomonic genetic aberration is an overexpression of BCL2 allowing proliferating B-cells to escape the positive and negative selection mechanisms for antibody specificity within the lymph node [4]. The majority of cases, 85%-90 harbor the translocation between t(14:18) creating a gene fusion of BCL2 and IGH. The mean age at diagnosis is 65 years, and the most common presenting symptom is painless lymphadenopathy [3]. This disease is less common in Europe and Asia, suggesting an environmental component. Follicular lymphoma typically follows an indolent course for several years with a ten-year risk of 28% transforming to diffuse large b-cell lymphoma, which carries a much poorer prognosis [5]. This transformation is often due to the acquisition of myelocytomatosis overexpression, thus resulting in the classical loss of tumor suppressor/gain of oncogene genetic pathogenesis [3].

Although the patient’s positive monospot test initially suggested infectious mononucleosis, the chronicity of symptoms and severity of purulent stomatitis are not characteristic of acute EBV infection, which typically resolves within weeks in immunocompetent young adults. The prolonged course in this case was instead driven by neoplastic B-cell proliferation, ultimately confirmed as nontranslocation follicular lymphoma.

We believe the most instructive aspect of this patient’s case was the delay in diagnosis and subsequent nutritional and infectious morbidity. His delay was primarily driven by labs and imaging consistent with Mononucleosis. However, as time passed, it became clear from both his stomatitis and spontaneous genital infections of *C. albicans* and *S. aureus* that this patient was immunocompromised. In this case, this ‘normal’ white blood cell level was likely secondary to lymphocyte neoplasia. The white blood cell differential within a complete blood count is frequently ignored in clinical practice. Deeper examination of the magnitude and pattern of white blood cell subtype response can help lead clinicians to faster, more accurate diagnosis of disorders involving the immune system.

Worldwide incidence of EBV infection is approximately 90%–95%, most occurring in childhood [6]. The sensitivity and specificity of monospot testing varies depending on the study. However, it is generally accepted that although there are a significant number of false negative tests, false positives are quite rare [7, 8]. Monospot testing evaluates for heterophile antibodies that bind to components of equine blood products or latex, not the virus itself. These antibodies are produced as a by-product of EBV driving B-cell growth and inhibiting apoptosis creating a permissive environment for development of heterophiles. Thus, although the majority of cases of monospot positivity are due to EBV, there is a small, nonzero number of positive test results that are due to heterophiles produced by other B-cell pathologies, including Systemic Lupus Erythematosus, human immunodeficiency virus, and *Brucella microti* [7–10]. It is worth noting that in our patient, his nontranslocation lymphoma presumably left his immunoglobulin genes intact to participate in the formation of heterophiles.

We hope this rare case presentation was of interest to the editors and readers of this journal and encourages providers to peruse the white blood cell differential on routine complete blood counts, and to occasionally interrogate the mechanism of action of common tests we all often take for granted.
